# Elevated serum platelet count inhibits the effects of brain functional changes on cognitive function in patients with mild cognitive impairment: A resting-state functional magnetic resonance imaging study

**DOI:** 10.3389/fnagi.2023.1088095

**Published:** 2023-03-27

**Authors:** Yuechan Zhang, Jing Liu, Zijun Wei, Jianing Mei, Qianqian Li, Xiaomin Zhen, Yunyun Zhang

**Affiliations:** ^1^Department of Neurology, Yueyang Hospital of Integrated Traditional Chinese and Western Medicine, Shanghai University of Traditional Chinese Medicine, Shanghai, China; ^2^Department of Pharmacy, Yueyang Hospital of Integrated Traditional Chinese and Western Medicine, Shanghai University of Traditional Chinese Medicine, Shanghai, China

**Keywords:** mild cognitive impairment, PLT, brain functional remodeling, cognitive function, moderating effect, rs-fMRI

## Abstract

**Objective:**

Brain function remodeling has been observed in patients with mild cognitive impairment (MCI) and is closely associated with cognitive performance. However, it is not clear if this relationship is influenced by complete blood counts. This study investigated the role of complete blood counts in the relationship between brain function and cognitive performance.

**Methods:**

Twenty-two MCI patients and eighteen controls were enrolled. All subjects underwent resting-state functional magnetic resonance imaging. A neuropsychological battery [Mini-Mental Status Examination, Auditory Verbal Learning Test (AVLT), Symbol Digit Modalities Test, Boston Naming Test (BNT), Shape Trails Test B (STT-B), Rey Complex Figure Test (RCFT), Hamilton Anxiety Rating Scale (HAMA), and Hamilton Depression Scale] was used to assess cognitive function, and MCI patients received complete blood counts tests for red blood cells (RBC), white blood cells, hemoglobin (HGB), monocytes, and platelet counts (PLT).

**Results:**

Compared with controls, MCI patients demonstrated significantly decreased amplitude of low-frequency fluctuation (ALFF) values in the left dorsolateral superior frontal gyrus, left post orbitofrontal cortex, right medial superior frontal gyrus, right insula, and left triangular inferior frontal gyrus. In the MCI group, there were associations between ALFF values of the left hippocampus (HIP.L) and AVLT (*p* = 0.003) and AVLT-N5 scores (*p* = 0.001); ALFF values of the right supramarginal gyrus (SMG.R) and BNT scores (*p* = 0.044); ALFF values of the right superior temporal gyrus (STG.R) and BNT scores (*p* = 0.022); ALFF values of the left precuneus (PCUN.L) and STT-B time (*p* = 0.012); and ALFF values of the left caudate nucleus (CAU.L) and RCFT-time (*p* = 0.036). Moreover, the HAMA scores were negatively correlated with RBC and HGB levels, and positively correlated with monocyte count. The PLT count was positively correlated with STT-B time. Additionally, high PLT count inhibited the effect of ALFF values of the PCUN. L on STT-B performance in MCI patients (*p* = 0.0207).

**Conclusion:**

ALFF values of the HIP. L, SMG.R, STG. R, PCUN.L, and CAU. L were associated with decreased memory, language, executive function, and visuospatial ability in MCI patients. Notably, elevated PLT count could inhibit the effect of brain functional changes in the PCUN.L on executive function in MCI patients.

## 1. Introduction

With the continual aging of the population, Alzheimer’s disease (AD) has become a major public health problem that imposes a heavy burden on families and society (Wolters and Ikram, 2018). Mild cognitive impairment (MCI) is an intermediate state between normal cognition and dementia, in which patients are more likely to suffer memory loss exceeding age-related cognitive decline but do not meet the diagnostic criteria for dementia (Petersen et al., 2001). MCI has a relatively high prevalence of 9.6–21.6% (Lara et al., 2016; Limongi et al., 2017; Jia et al., 2020). In addition, MCI may be a risk factor for dementia in later life. The annual and 5-year conversion rates of MCI to dementia were reported as 12 and 72%, respectively (Petersen, 2004; Inui et al., 2017). A growing number of studies are now focusing on MCI.

Although the pathogenetic mechanisms of MCI are poorly defined, rapid progress in neuroimaging has provided imaging markers that could help elucidate the mechanisms (Li W. et al., 2021). Resting-state functional magnetic resonance imaging (rs-fMRI) is an imaging technique that induces changes in the nuclear magnetic signal through alterations of the blood oxygen level, and has been widely used in research on various neuropsychiatric diseases such as stroke (Baldassarre et al., 2016), Parkinson’s disease (PD) (Tessitore et al., 2019), AD (Colangeli et al., 2016), and depression (Kaiser et al., 2015). The amplitude of low-frequency fluctuation (ALFF) reflects alterations in regional brain activity and has been employed in studies of MCI (Cai et al., 2017; Lin et al., 2018). Previous rs-fMRI studies demonstrated brain functional remodeling in MCI patients. Compared with healthy subjects, ALFF values in the bilateral hippocampus, bilateral fusiform gyrus, bilateral parahippocampal gyrus, and left lingual gyrus were significantly higher in MCI patients (Long et al., 2016). Other studies found that ALFF values in the posterior cingulate gyrus of MCI patients were lower than those of controls (Bai et al., 2008). Consequently, fMRI is advantageous for exploring brain function in MCI.

Brain function is closely associated with cognitive function. Recent rs-MRI studies showed that ALFF values in the right insula/superior temporal gyrus were decreased in MCI patients, which was related to cognitive performance in the verbal fluency test of the Montreal Cognitive Assessment—Basic (Guo et al., 2022). In people with vascular MCI, the regional homogeneity of the bilateral cingulate cortex was negatively correlated with the Montreal Cognitive Assessment score (Zhuang et al., 2021). In early PD patients with MCI, the ALFF in the opercular part of the right inferior frontal gyrus was negatively correlated with semantic fluency and negatively correlated with processing speed attention (Wang et al., 2018). However, the effects of changes in regional functional brain activity in MCI patients on specific cognitive domains have been poorly studied. Moreover, although brain function is associated with cognitive function in MCI patients, it is unclear whether the relationship is influenced by other factors.

Cognitive function is influenced by complete blood counts. Among 1,227 elderly subjects, serum hemoglobin (HGB) levels were inversely associated with the risk of cognitive impairment, and it was suggested that low hemoglobin concentrations may predict the development of cognitive dysfunction (Trevisan et al., 2016). A cross-sectional study found that increased red blood cell (RBC) distribution width was associated with poorer verbal memory and attentional function in middle-aged people (Beydoun et al., 2020). Moreover, Platelet count (PLT) was negatively associated with the cognitive function scores of patients with atrial fibrillation (Sun et al., 2021). rs-MRI uses blood-deoxygenated hemoglobin as an endogenous contrast agent to evaluate oxygen availability. In addition to the inhomogeneity and intensity of the magnetic field, applied pulse sequence, and vasoconstriction, factors such as hematocrit and blood volumes are also very important and influential. The available evidence suggests that, as well as brain function and cognitive performance, the complete blood counts and cognitive function are also related. We hypothesized that the complete blood counts influence the relationship between brain function and cognitive performance in MCI patients. Elucidating this relationship will shed light on the role of the complete blood counts in brain function and cognitive performance, and may provide new strategies for diagnosing and treating MCI using rs-fMRI.

We collected neuropsychological and rs-MRI data from 40 subjects, and obtained the complete blood counts of 22 MCI patients, to explore the effect of complete blood counts on the relationship between function and cognitive performance.

## 2. Methods

### 2.1. Participants

From January 2022 to October 2022, 22 MCI patients (9 males and 13 females aged 66.14 ± 6.45 years) and 18 healthy controls (6 males and 12 females aged 63.78 ± 7.08 years) were enrolled from the Yueyang Hospital of Integrated Traditional Chinese and Western Medicine affiliated to Shanghai University of Traditional Chinese Medicine. This case–control study was approved by the Institutional Review Board of the Yueyang Hospital of Integrated Traditional Chinese and Western Medicine (No. 2021–138). All subjects were informed about the study and signed the informed consent form prior to the experiments.

Inclusion criteria were as follows: (1) aged 50–75 years both genders; (2) cognitive complaints within the last year; and (3) met the following established diagnostic criteria for MCI (Winblad et al., 2004): ① cognitive impairment reported by the patients themselves or close associates, or observed by clinicians; ② objective evidence of impairment in one or more cognitive domains; ③ potential mild impairment of complex instrumental daily activities but the ability to perform independent daily activities; and ④ not satisfying the criteria for a diagnosis of dementia. Exclusion criteria for MCI were as follows: (1) history of acute cerebral vascular disease in the past 3 months; (2) history of cerebral hemorrhage, cerebral infarction (infarct diameter ≥ 15 mm), nervous system tumor, immune brain injury, or hypothyroidism; (3) taking medication to improve cognitive function within the past 3 months; (4) contraindications for MRI such as claustrophobia or metallic implants; (5) history of mental illness such as severe depression or anxiety; (6) hearing or speech disorder precluding compliance with the study requirements; and (7) diabetes, malignant tumors, or a serious heart, liver, kidney, or hematopoietic system disease.

Eighteen HCs were recruited from the same sources and matched with the MCI subjects in terms of age, gender, and education level. The inclusion criteria for the HC group were as follows: (1) aged 50–75 years, both genders; (2) no cognitive complaints; and (3) a neuropsychological evaluation score below the threshold for impairment. The exclusion criteria were the same as for the MCI subjects.

### 2.2. Neuropsychological tests

Overall cognitive function was assessed by the Mini-Mental Status Examination (MMSE) (Folstein et al., 1975). The Auditory Verbal Learning Test (AVLT) (Guo et al., 2009) was used to evaluate episodic memory. AVLT includes five trials of the recall of a 12-word list and measures immediate recall, short recall, and long recall of memory. Over five trials, the number of correctly recalled words was recorded as AVLT scores, and the scores of AVLT-N5 were the number of correctly recalled words in the long-delayed recall of memory. The Symbol Digit Modalities Test (SDMT) (Smith, 1982) was used to test working memory and the speed of information processing. We assessed language and executive function using the Boston Naming Test (BNT) (Cheung et al., 2004) and Shape Trails Test B (STT-B) (Zhao et al., 2013), respectively. The Rey Complex Figure Test (RCFT) (Loring et al., 1990) was used to assess visuospatial structural ability and nonverbal memory, and copying time and scores were recorded. Finally, we used the Hamilton Anxiety Rating Scale (HAMA) (Hamilton, 1959) and Hamilton Depression Scale (HAMD) (Hamilton, 1960) to assess anxiety and depression symptoms, respectively. Two trained neurologists conducted the neuropsychological evaluation and the clinical diagnosis of MCI. In cases where the diagnosis was inconsistent, a third experienced neurologist made the final diagnosis.

### 2.3. Serum index

In the MCI group, blood samples were collected in the morning after the subjects had fasted for 12 h. The BC-6800PLUS automatic blood cell analyzer (Mindray, Shenzhen, China) was used to obtain the RBC count, white blood cells (WBC) count, HGB level, monocyte count, and PLT count. Blood samples were analyzed within the clinical laboratory of the hospital and laboratory professionals performed all procedures in strict accordance with standard procedures.

### 2.4. MRI data acquisition

MRI data of all subjects were acquired on a 3.0 T scanner (Siemens AG, Erlangen, Germany) with a 32-channel phased array head coil. While lying on the scanner bed in a supine position and wearing 3 M noise-reducing earplugs, the subjects were instructed to close their eyes, remain relaxed, and avoid cognitive or motor activity during the scan. The rs-fMRI data were obtained using a single-pass gradient recalled echoplanar imaging sequence with the following parameters: interleaved scanning order; slice number = 43, matrix size = 64 × 64; field of view = 230 mm× 230 mm; repetition time = 3,000 ms; flip angle = 90°; slice thickness = 3.0 mm; gap = 0 (voxel size = 3.6 mm× 3.6 mm × 3.0 mm); and a number of acquisitions = 200.

### 2.5. Data processing

Functional images of each subject were obtained using the Resting-State Functional Magnetic Resonance Imaging Data Analysis Toolkit (REST; version 1.8) (Song et al., 2011) using the SPM12 toolbox (Wellcome Centre for Human Neuroimaging^1^), which was run through MATLAB (version 2013b; MathWorks, Natick, MA, United States). Data preprocessing and ALFF analysis were performed using a previously published method (Li L. et al., 2021). Quality control included field and motion correction, co-registration, and spatial normalization. Subjects were excluded if the head movement amount was ≥2.5 mm or rotation was ≥2.5°. Nuisance variables in the regression included white matter, cerebrospinal fluid, and six head motion parameters.

### 2.6. Statistical analysis

SPSS 25.0 statistical software was used for data analysis. Normally distributed quantitative data are expressed as mean ± standard deviation, and the t-test was used for group comparisons. Data that did not conform to a normal distribution are expressed as median and were compared between groups using the nonparametric rank-sum test. Qualitative data are presented as percentages and intergroup comparisons were performed using the chi-square test. Pearson’s correlation was used to analyze correlations among brain function, serum indicators, and neuropsychological scores. We also analyzed potential moderators of the relationship between brain function and cognitive function using the PROCESS macro for SPSS. The fMRI images were segmented into 90 regions (excluding the cerebellum) according to the Automated Anatomical Labeling system (Tzourio-Mazoyer et al., 2002). The average ALFF value of each region of interest was extracted for the Pearson correlation and modulatory effect analyses. All statistical tests were two-sided and *p*-values <0.05 were considered statistically significant.

The ALFF maps were compared between two groups. The two-sample t-test was used for the two groups. AlphaSim correction was used for multiple comparisons, which was performed using the REST plus toolkit. The resulting statistical map was set at *p* < 0.05 (AlphaSim correction) with a combined individual voxel *p* < 0.001.

## 3. Results

### 3.1. Demographic characteristics

Demographic and clinical characteristics of the MCI and HC groups are shown in Table 1. The two groups did not differ significantly in age, gender, body mass index, or education level (*p* > 0.05). However, compared with the HC group, the MMSE, AVLT, AVLT-N5, SDMT, RCFT, and BNT scores of the MCI patients were lower and the time to complete the STT-B was longer (*p* < 0.05). There were no significant group differences in any other parameters (*p* > 0.05).

**Table 1:** Demographic and neuropsychological data of the MCI and HC groups.

Characteristics	HC (*n* = 18)	MCI (*n* = 22)	*P*
Gender (male:female %)	6:12 (33.33:66.67)	9:13 (40.91:59.09)	0.622^a^
Age (*y*)	63.78 ± 7.08	66.14 ± 6.45	0.278^b^
Education (*y*)	10.78 ± 2.29	9.91 ± 2.58	0.272^b^
BMI (kg/m^2^)	23.35 ± 2.81	23.16 ± 2.63	0.826^b^
Handedness (R:L %)	17:1 (94.44:5.56)	22:0 (100.00:0.00)	0.919^a^
MMSE scores	28.17 ± 1.47	26.55 ± 1.82	0.004^b^
AVLT scores	34.72 ± 6.66	20.50 ± 6.78	<0.0001^b^
AVLT-N5 scores	7.00 ± 1.71	3.14 ± 1.98	<0.0001^b^
SDMT scores	44.61 ± 9.47	21.27 ± 9.62	<0.0001^b^
STT-B (s)	110.99 ± 29.10	199.93 ± 83.30	<0.0001^b^
RCFT-time (s)	154.17 ± 50.72	198.71 ± 85.21	0.058^b^
RCFT scores	32.11 ± 3.13	29.05 ± 4.56	0.020^b^
BNT scores	25.39 ± 1.50	20.91 ± 3.64	<0.0001^b^
HAMA scores	3(1.75,3.25)	3(2,5)	0.448^c^
HAMD scores	0.5(0,1.25)	1(0,4)	0.257^c^

^a^the chi-square test; ^b^the two-sample *t*-test; ^c^the nonparametric rank-sum test. HC, healthy control; MCI, mild cognitive impairment; MMSE, mini-mental status examination; AVLT, auditory verbal learning test; AVLT-N5, auditory verbal learning test – long delay recall; SDMT, symbol digit modalities test; STT-B, shape trails test B; RCFT, Rey complex figure test; BNT, Boston naming test; HAMA, Hamilton anxiety rating scale; HAMD, Hamilton depression scale.

### 3.2. Comparison of brain function between the MCI and HC groups

**Figure 1:**
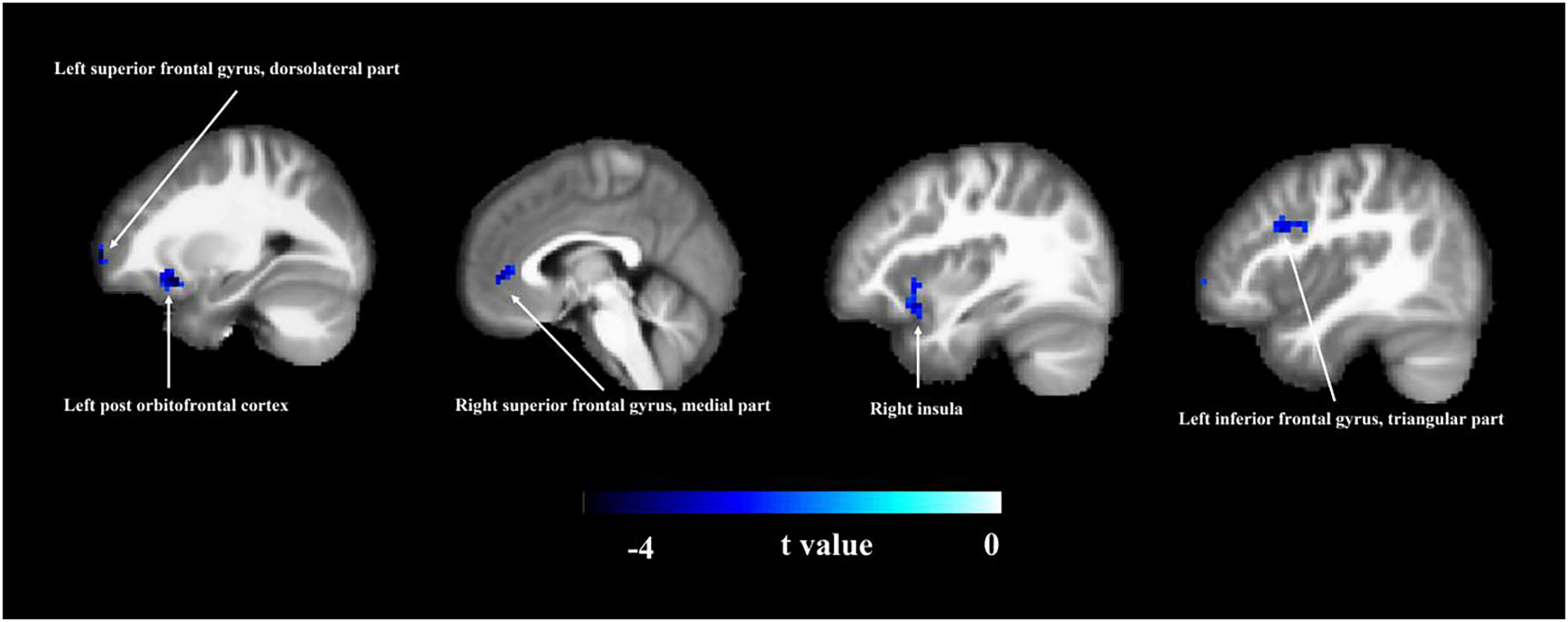
Amplitude of low-frequency fluctuation (ALFF) analysis. Brain region information of ALFF was compared between MCI and HC groups. The two-sample *t*-test was used to compare the groups. Areas in blue indicate significantly decreased ALFF values. Compared with the HCs, the MCI patients showed significantly decreased ALFF values in the left dorsolateral superior frontal gyrus, left post orbitofrontal cortex, right medial superior frontal gyrus, right insula, and left triangular inferior frontal gyrus. MCI, mild cognitive impairment; HC, healthy control.

As shown in Figure 1 and Table 2, there were significant differences in ALFF values between the MCI patients and HC groups. The MCI patients had significantly decreased ALFF values in the left dorsolateral superior frontal gyrus, left post orbitofrontal cortex, right medial superior frontal gyrus, right insula, and left triangular inferior frontal gyrus (*p* < 0.05).

**Table 2:** Brain region information of ALFF comparison between patients with MCI and HC groups.

Brain regions	Cluster size	Cluster centroid MNI coordinates			*t*-value
		*x*	*y*	*z*	
Left superior frontal gyrus, dorsolateral part	35	−30	66	3	−4.520
Left post orbitofrontal cortex	35	−27	21	−15	−4.351
Right superior frontal gyrus, medial part	28	3	45	0	−3.791
Right insula	26	36	18	−15	−3.484
Left inferior frontal gyrus, triangular part	49	−42	15	27	−3.382

### 3.3. Relationship between brain function and cognitive function in patients with MCI

**Figure 2:**
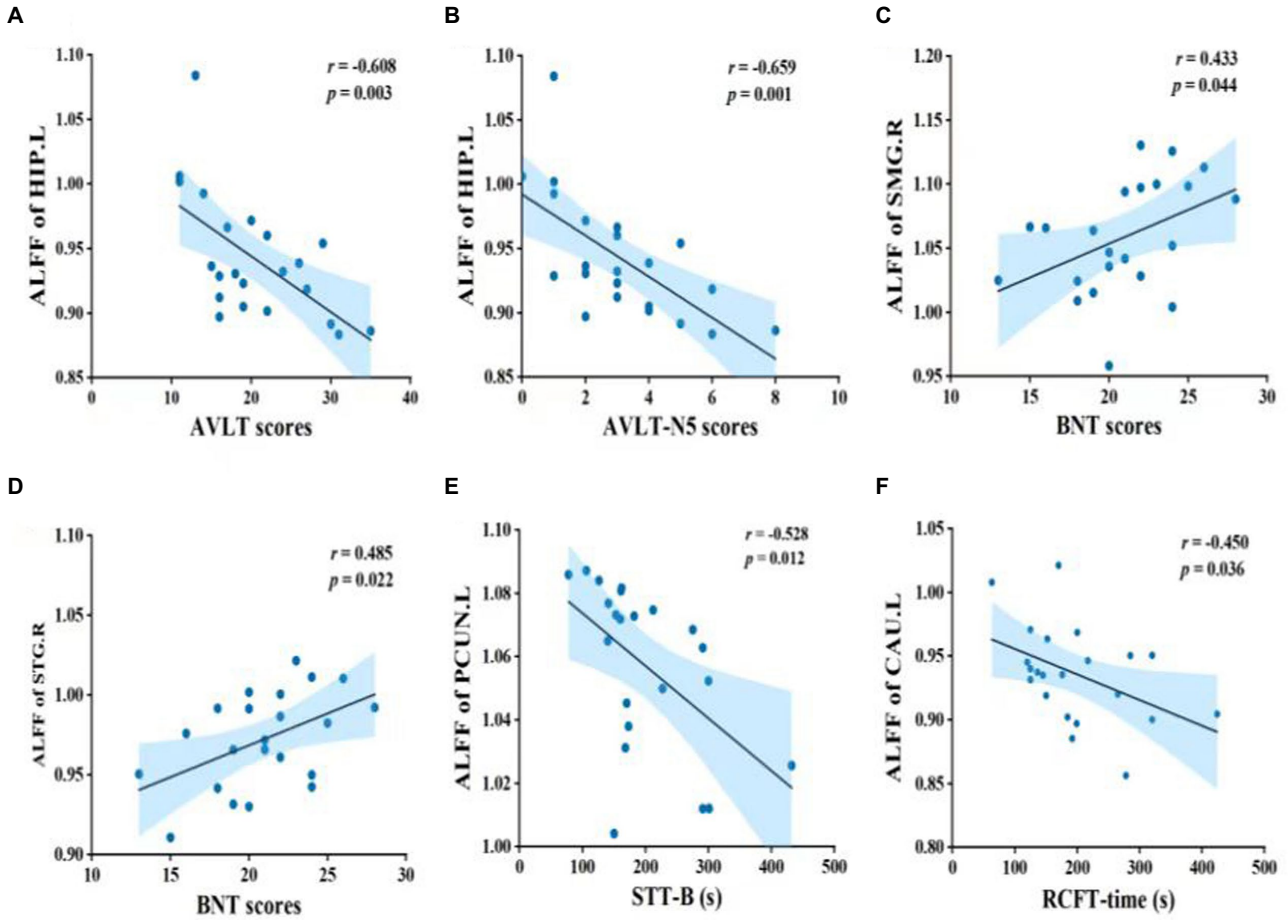
Pearson’s correlation between brain function and cognitive performance in MCI patients. Abbreviations: ALFF, amplitude of low-frequency fluctuation; HIP.L, left hippocampus; AVLT, auditory verbal learning test; AVLT-N5, auditory verbal learning test – long delay recall; SMG.R, right supramarginal gyrus; BNT, Boston naming test; STG.R, right superior temporal gyrus; PCUN.L, left precuneus; STT-B, shape trails test B; CAU.L, left caudate nucleus; RCFT, Rey complex figure test; MCI, mild cognitive impairment.

Pearson’s correlation was used to measure the strength of the association between brain activity and cognitive function in the MCI group (Figure 2). ALFF values of the left hippocampus (HIP.L) were negatively correlated with AVLT and AVLT-N5 scores. Moreover, there were significant associations between ALFF values of the right supramarginal gyrus (SMG.R) and BNT scores; ALFF values of the right superior temporal gyrus (STG.R) and BNT scores; ALFF values of the left precuneus (PCUN.L) and STT-B time; and ALFF values of the left caudate nucleus (CAU.L) and RCFT-time.

### 3.4. Relationship between complete blood counts and cognitive function in patients with MCI

**Figure 3:**
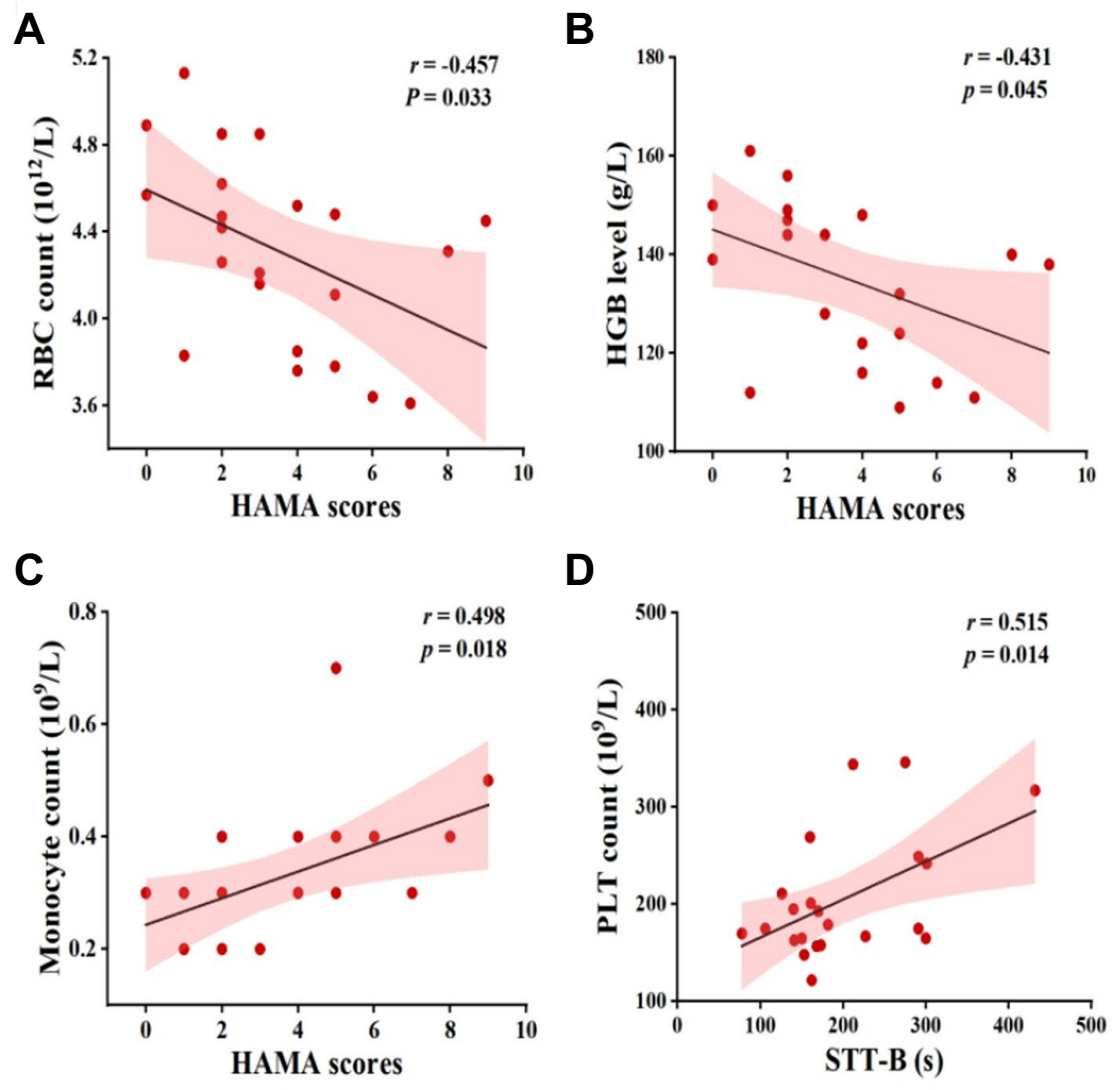
Pearson’s correlation between complete blood counts and cognitive function in MCI patients. Abbreviations: RBC, red blood cells; HGB, hemoglobin; HAMA, Hamilton anxiety rating scale; PLT, platelet; STT-B, shape trails test B; MCI, mild cognitive impairment.

As also illustrated in Figure 3, a lower RBC count and HGB level were associated with higher HAMA scores. Specifically, the monocyte count was positively correlated with the HAMA scores and the PLT count was positively correlated with the STT-B time. There were no significant correlations between any other blood counts and cognitive performance metrics (*p* > 0.05).

### 3.5. Serum PLT inhibited the effects of brain functional changes on cognitive function in MCI patients

**Figure 4:**
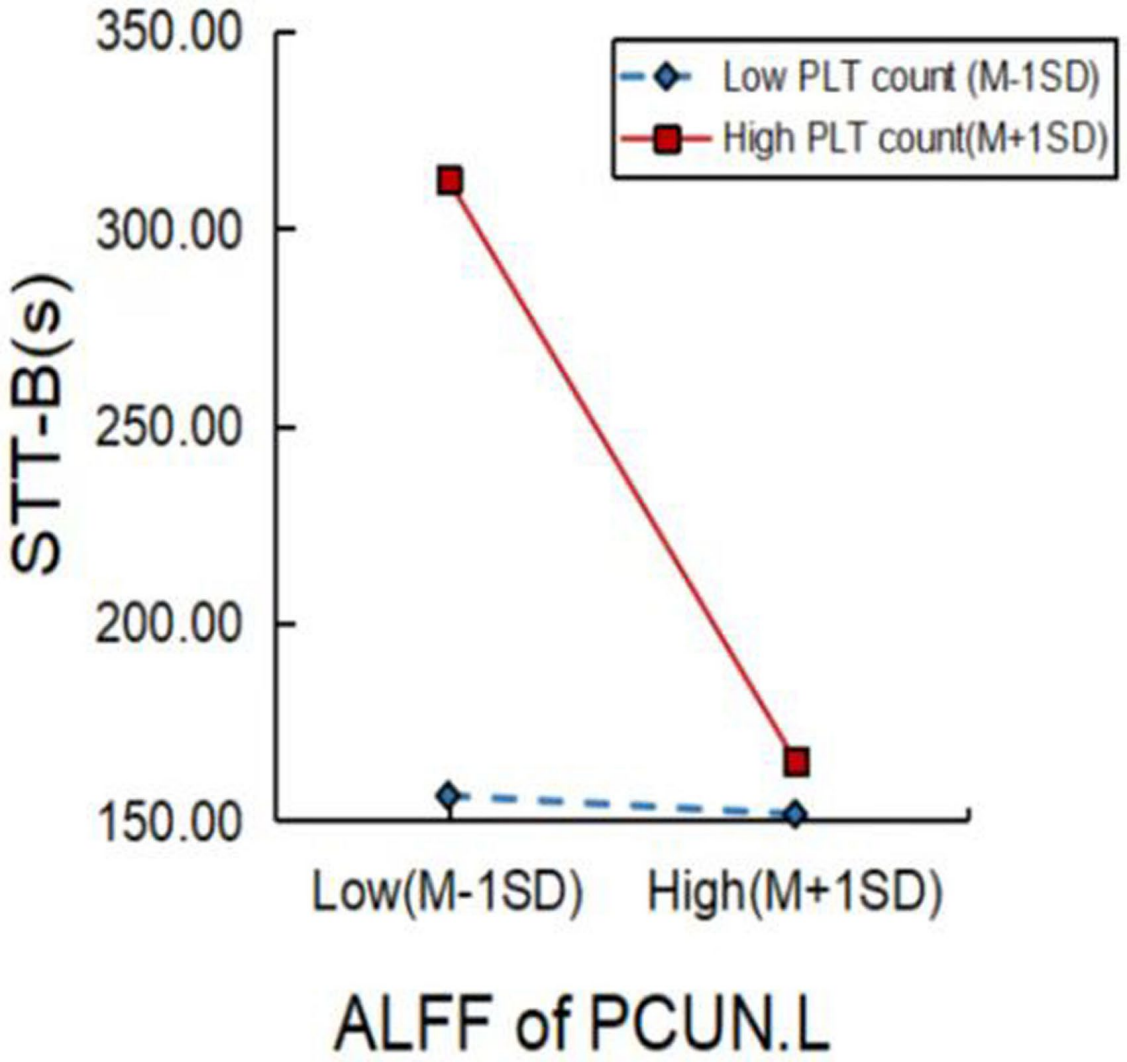
Platelet (PLT) count significantly moderated the relationship between brain function and cognitive performance in MCI patients. PLT, platelet; ALFF, amplitude of low-frequency fluctuation; PCUN.L, left precuneus; STT-B, shape trails test B; MCI, mild cognitive impairment.

We further explored factors moderating the relationship between brain activity and cognitive function. As shown in Figure 4, a higher PLT count significantly moderated the relationship between ALFF values of the PCUN.L and STT-B performance (*p* = 0.0207).

## 4. Discussion

Mild cognitive impairment is considered to be a “transition state” between normal cognitive functioning and dementia, and the main clinical manifestation of early-stage MCI is memory decline. At present, the best approach for diagnosing MCI is to use memory tests in combination with comprehensive neuropsychological tests related to other cognitive domains (Belleville et al., 2017). In our study, in addition to global cognitive decline, patients with MCI exhibited decreased episodic memory, executive function, verbal fluency, and visuospatial ability. Previous studies have shown that patients with MCI experience deficits in episodic and semantic memory (Dudas et al., 2005). Compared with elderly controls, MCI patients exhibited impairments in episodic memory and executive function (Eliassen et al., 2017). Cross-sectional and prospective studies of preclinical AD and MCI found that MCI patients initially experienced impairment in verbal episodic memory, followed by visual episodic memory (Celsis, 2000). Therefore, close attention should be paid to the memory, executive, language, and visuospatial abilities of MCI patients to prevent the progression to dementia.

In our study, MCI patients showed significantly decreased ALFF values in the left dorsolateral superior frontal gyrus, left post orbitofrontal cortex, right medial superior frontal gyrus, right insula, and left triangular inferior frontal gyrus. We found that the MCI-pathology-related brain regions were mainly concentrated in the frontal lobe. As our patients were in the early stage of cognitive impairment, the hippocampus may have been capable of compensating for some loss of function. Besides, the prefrontal lobe participates in more advanced cognitive functions, and it plays important roles in learning, memory, thinking, and other advanced functions (Fan et al., 2014; Pezzulo et al., 2018). Previous studies support our findings. For example, compared with HCs, the ALFF was decreased in the right superior frontal gyrus and right middle frontal gyrus of patients with amnestic mild cognitive impairment (aMCI) (Liu et al., 2022). Another study found that patients with aMCI had decreased ALFF values in the posterior cingulate/precuneus, medial prefrontal cortex, hippocampus/ parahippocampal gyrus, basal ganglia, and prefrontal regions, while ALFF values in several regions of the occipital and temporal lobe were higher compared with the NC group (Han et al., 2011). Studying the changes of brain function that occur in MCI patients, and their relationships with performance in various cognitive domains will help us to further clarify the mechanisms of MCI.

Hippocampus is considered central to cognitive functions related to learning and memory. Hippocampal atrophy occurs before clinical signs of AD appear and has proven to be one of the most informative and convenient biomarkers of AD (Janocko et al., 2012; de Flores et al., 2015). In a previous study, the inferior and anterior inferior hippocampal volumes in MCI patients were associated only with immediate recall (Carlesimo et al., 2015), while another study reported that the ALFF values of the bilateral hippocampus and bilateral parahippocampal gyrus were significantly higher in MCI patients than controls (Long et al., 2016). In this study, ALFF values of the HIP.L were inversely related to episodic memory. Hence, increased ALFF values of the HIP.L may impair the function of episodic memory in MCI patients. Wernicke’s area contains the supramarginal gyrus, which is associated with speech and language comprehension (Resende Resende et al., 2020). Moreover, the superior temporal gyrus is responsible for auditory processing and speech reception (Ding et al., 2016). Functional connectivity of the posterior cingulate with the STG.R, bilateral middle temporal gyrus, and bilateral lingual gyrus was significantly reduced in MCI patients (Wu et al., 2013). The decreased ALFF values in the SMG.R and STG.R seen in our MCI patients might reflect impaired verbal fluency. Moreover, the precuneus is associated with many high-level cognitive functions, especially self-relevant information processing (Uka, 2016). Our study revealed that decreased ALFF in the PCUN.L was correlated with a decline in executive function. The caudate nucleus, which is part of the striatum, directs attention and behavior in concert with the brain’s learning and memory system (Huang et al., 2021). Reduced [^18^F]-fluorodeoxyglucose uptake in the caudate nucleus in PD patients was implicated in impairments in verbal fluency, working memory, and attentional functioning (Rinne et al., 2000). We found that ALFF values of the CAU.L were related to the RCFT-time, suggesting that dysfunction of the CAU.L might impact the visuospatial ability and nonverbal memory of patients with MCI.

The RBC count and HGB level were negatively correlated with anxiety in our MCI group. No study has analyzed the correlations among the RBC count, HGB level, and anxiety in MCI patients. A cohort study including 2,920 participants found no independent associations between depression and/or anxiety disorders with HGB level or anemia status (Lever-van Milligen et al., 2014), while another study found that maternal anxiety was not only related to pregnancy, low education level, and low family income, but also a low HGB level (Begum and Biswas, 2021). In a study of 9,274 participants, RBC count and mean corpuscular hemoglobin level were negatively associated with depression/anxiety symptoms (Shafiee et al., 2017). The relationships among the RBC count, HGB level, and anxiety may vary depending on the characteristics of the study population, such as their diagnoses and ethnicity. Therefore, further study is required. Also, we found a positive correlation between the monocyte count and anxiety in MCI patients. Monocytes are an important component of the body’s defense system and play a role in the immune response. In pathological conditions, especially stress-related ones, activated glial cells and peripheral blood monocytes accumulate in the brain, which exacerbates anxiety and depression symptoms (Wohleb and Delpech, 2017; McKim et al., 2018). For example, parents of children with cancer exhibited higher peripheral blood monocyte count compared with parents of healthy children (Agbayani et al., 2022). Although our findings suggested monocytes were associated with anxiety, considering the small sample size of this study, the effect of monocytes on anxiety symptoms of MCI patients still needs to be further determined.

Platelets are produced by megakaryocytes in the hematopoietic tissue of the bone marrow (Cardigan et al., 2005). In addition to their roles in clotting, hemostasis, and the repair of damaged blood vessels, PLT count is also involved in the regulation of immunity and inflammation (Kim et al., 2018). When activated, PLT can release a variety of bioactive factors, including cytokines, chemokines, and neurotransmitters (Qureshi et al., 2009). Activated PLT expresses large amounts of amyloid precursor protein and release beta-amyloid, which attaches to blood vessel walls and plays an important role in plaque deposition in the Alzheimer’s brain (Humpel, 2017; Shi et al., 2017). Furthermore, *β*-enzyme activity in the PLT membrane is elevated in AD and MCI patients (McGuinness et al., 2016). In addition, PLT activity was found to be an independent predictor of the severity of vascular cognitive impairment (Stellos et al., 2014). Similar to previous studies, we found that the PLT count in MCI patients had an inverse relationship with cognitive function. Notably, we found that PLT moderated the relationship between brain activity and cognitive function in MCI patients, and an elevated PLT count inhibited the effect of functional changes in the PCUN.L on executive function. This result implies that the PLT count in MCI patients can serve as a biological marker of brain activity that may affect cognitive behavior.

## 5. Limitations

This exploratory study had some limitations. First, the sample size was small, which limited the statistical power. Second, we only collected peripheral blood cells from MCI patients and did not analyze PLT derivatives or the cerebrospinal fluid; doing so would have allowed for a more comprehensive assessment of potential brain activity biomarkers of cognitive impairment. Third, it might be more accurate to compare patients with different types of MCI and perform subgroup analyses.

## 6. Conclusion

We explored the role of complete blood counts in the relationship between brain function and cognitive performance in MCI patients and found that an elevated PLT count inhibited the effect of functional changes in the PCUN.L on executive function. Despite some limitations, the findings of this study improve our understanding of the neural mechanisms underlying MCI, and may inform new approaches to the diagnosis and treatment of this condition.

## Data availability statement

The raw data supporting the conclusions of this article will be made available by the authors, without undue reservation.

## Ethics statement

The studies involving human participants were reviewed and approved by the ethics committee of Yueyang Hospital of Integrated Traditional Chinese and Western Medicine. The patients/participants provided their written informed consent to participate in this study. Written informed consent was obtained from the individual(s) for the publication of any potentially identifiable images or data included in this article.

## Author contributions

YCZ and JL performed the experiments, analyzed the data, and drafted the manuscript. ZW and JM performed the experiments and collected the data. QL processed the data. XZ and YYZ designed the study, reviewed the manuscript, and approved the final version. All authors contributed to the article and approved the submitted version.

## Funding

This study was supported by the Shanghai Science and Technology Committee (Grant No. 22Y11920700), Shanghai “Rising Stars of Medical Talent” Youth Development Program [Grant No. SHWRS (2020)_087] and Project of Yueyang Hospital of Integrated Traditional Chinese and Western Medicine, Shanghai University of Traditional Chinese Medicine (Grant No. 2018YJ06).

## Conflict of interest

The authors declare that the research was conducted in the absence of any commercial or financial relationships that could be construed as a potential conflict of interest.

## Publisher’s note

All claims expressed in this article are solely those of the authors and do not necessarily represent those of their affiliated organizations, or those of the publisher, the editors and the reviewers. Any product that may be evaluated in this article, or claim that may be made by its manufacturer, is not guaranteed or endorsed by the publisher.

## Footnotes

^1^ http://www.fil.ion.ucl.ac.uk/spm
